# Evaluation of antibodies against human HSP60 in patients with MPO-ANCA associated glomerulonephritis: a cohort study

**DOI:** 10.1186/1740-2557-3-4

**Published:** 2006-05-05

**Authors:** Marjan C Slot, Ruud Theunissen, Pieter van Paassen, Jan GMC Damoiseaux, Jan Willem Cohen Tervaert

**Affiliations:** 1 Dept. of Clinical and Experimental Immunology, university hospital Maastricht, P.O. Box 5800, 6202 AZ Maastricht, The Netherlands

## Abstract

**Background:**

Human Heat Shock Protein 60 (hHSP60) has been implicated in autoimmunity through molecular mimicry, based on the high degree of homology with HSP65 of micro-organisms leading to autoimmune recognition of the human protein. Additionally, sequence homology between hHSP60 and myeloperoxidase (MPO) has been described. MPO is a major autoantigen in vasculitis associated with antineutrophil cytoplasmic antibodies (ANCA). We hypothesized that infections may trigger the ANCA response against MPO through hHSP60.

**Methods:**

In 86 consecutive patients with ANCA-associated vasculitis (AAV), anti-hHSP60 and anti-mycobacterial HSP65 were measured by ELISA. Patients were compared with 69 healthy controls (HC). Continuous data between groups were compared using Wilcoxon signed rank test and Kruskal-Wallis test with Dunn's post-test when appropriate. Correlations between data were derived using Spearman correlation. Odds ratios and 95% confidence intervals were obtained using Fisher's exact test.

**Results:**

At diagnosis, median anti-mHSP65 level was higher in AAV (median [range]: 42.5 [0–500]), and subsequently in MPO-ANCA (44 [7–500]), compared to HC (22 [0–430]). Anti-hHSP60 levels in AAV were not higher compared to HC (18 [0–319] and 18.5 [0–98], respectively). However, in MPO-ANCA anti-hHSP60 levels were increased (32.5 [0–319]) compared to PR3-ANCA (13 [0–79]) and HC. We could not detect cross-reactivity between hHSP60 and MPO-ANCA. There was a correlation between anti-mHSP65 and anti-hHSP60 levels (r = 0.32, *P *= 0.003) but not between anti-hHSP60 and MPO-ANCA (r = -0.064, *P *= 0.69).

**Conclusion:**

Antibodies against mHSP65 are higher in AAV compared to HC, and anti-hHSP60 antibodies are higher in patients with MPO-ANCA than in patients with PR3-ANCA and HC. Although this finding may be indicative for cross-reactivity between MPO-ANCA and hHSP60, additional assays did not support this hypothesis.

## Introduction

Small vessel vasculitides, such as Wegener's granulomatosis (WG) and microscopic polyangiitis (MPA), are strongly associated with antineutrophil cytoplasmic antibodies (ANCA), which are either directed to myeloperoxidase (MPO) or proteinase 3 (PR3) [1-3]. These diseases can occur in any organ system but the respiratory tract and the kidneys are most frequently involved. Untreated, WG results in death within weeks to months. Since the introduction of cyclophosphamide and prednisolone as standard treatment, survival has improved dramatically from less than 20% at 1 year reported in 1958 [4] to at least 60% 5-years survival reported in the past ten years [5-8].

The mechanism by which ANCA are induced is as yet unclear. Certain drugs have been linked to the induction of ANCA and the onset of ANCA-associated vasculitis (AAV) [9]. Recently, it has been described that an autoimmune response may be induced by the presence of a peptide that is antisense or complementary to the autoantigen, for instance, PR3 [10]. This immune response may induce anti-idiotypic antibodies (autoantibodies) that cross-react with the autoantigen. Another favored hypothesis is that infections may trigger an ANCA response [11]. The proposed mechanisms by which infections break self tolerance can include bystander damage, unveiling of 'hidden' self epitopes, determinant molecular spreading and molecular mimicry [11].

Heat Shock Protein (HSP) 65 is an immunodominant antigen in micro-organisms, and has already been implicated in the pathogenesis of vasculitides such as Kawasaki disease [12] and Behcet disease [13]. The human equivalent, hHSP60, has been implicated in autoimmunity through molecular mimicry, based on the high degree of homology with HSP65 of micro-organisms leading to autoimmune recognition of the human protein [14]. In its turn, HSP60 shares sequence homology with MPO [15]. Thus, infections may trigger the ANCA response against MPO through hHSP60. To test this hypothesis, we determined the presence of antibodies against hHSP60 and mycobacterial HSP65 (mHSP65) in patients with MPO-ANCA and compared them to patients with PR3-ANCA and healthy controls (HC). Results showed that antibodies against mHSP65 are higher in AAV compared to HC, and anti-hHSP60 antibodies are higher in patients with MPO-ANCA than in patients with PR3-ANCA and HC. However, additional assays did not support our hypothesis of cross-reactivity between MPO-ANCA and hHSP60.

## Methods

### Patients

We retrospectively identified all patients in our renal biopsy registry [16,17] diagnosed with crescentic glomerulonephritis between January 1977 and July 2003 without evidence of systemic lupus erythematosus, IgA nephropathy, Henoch Schönlein purpura, post-infectious glomerulonephritis or cryoglobulinaemia. Serum samples, taken at the time of biopsy, were tested for the presence of ANCA, according to a multistep procedure that combines indirect immunofluorescence with direct and capture enzyme-linked immunosorbent assays (ELISAs) [18]. We thus identified 109 patients with AAV with biopsy-proven renal involvement: 46 patients with PR3-ANCA only (42%), 49 patients with MPO-ANCA only (45%), 4 patients with both PR3- and MPO-ANCA (4%) and 10 patients with MPO-ANCA with the presence of anti-glomerular basement membrane antibodies (9%). Only patients who were single positive for PR3- or MPO-ANCA, and had enough serum available for all tests, were included (n = 86). Additionally, 40 healthy controls (HC) (laboratory personnel) were tested to determine the cut-off value for the anti-hHSP60 test and, 69 HC were tested to determine the cut-off value for the anti-mHSP65 test. This study was performed in accordance with the 1997 Declaration of Helsinki of the World Medical Association. Demographic data on patients and controls are presented in table 1.

**Table 1:** Demographic data on patients and healthy controls

	AAV (*N *= 86)	MPO-ANCA (*N *= 42)	PR3-ANCA (*N *= 44)	HC (*N *= 69)
Age (years)*	62.2 ± 13.5	65.9 ± 10.9	59.7 ± 15.2	40.4 ± 9.8
Male gender (%)	69	64	73	59

AAV, ANCA-associated vasculitis; MPO-ANCA, antineutrophil cytoplasmic antibodies against myeloperoxidase; PR3-ANCA, ANCA against proteinase 3; HC, healthy controls

* Mean ± standard deviation

### Sera

Serum samples, taken at the time of biopsy, were used to determine the presence of anti-hHSP60 and anti-mHSP65 in patients with AAV. These sera had been stored at -80°C, in some patients for over 20 years. However, when testing the antibody response, there was no difference in antibody level between patients included in 2003 and patients included in earlier years.

### ELISA for the detection of antibodies against human HSP60 and mycobacterial HSP65

Recombinant hHSP60 (Stressgen, Victoria, Canada) was diluted to 2 μg/mL in coating buffer containing 0.1 M NaHCO_3_^- ^(pH 9.6), and 50 μL/well (i.e., 0.1 μg/well) was incubated in wells of microplates (Nunc MaxiSorp™, Rochester, NY) overnight at 4°C. Control wells were incubated with coating buffer alone. Wells were then incubated overnight at 4°C with 100 μL/well coating buffer containing 1% grade V bovine serum albumin (BSA, Sigma, St. Louis, MO) to block non-specific binding. Plates were then washed 3 times with PBS containing 0.05% Tween 20 (PBS-T). Plates were preincubated at room temperature with 200 μL/well of preincubation buffer, containing 0.05% Tween 20 and 1% BSA in PBS, for 1 hour. After washing 2 times with PBS-T, wells were incubated in triplicates with patient serum in a previously established optimal dilution of 1:50, 50 μL/well, in incubation buffer and kept overnight at 4°C. The next day, plates were washed 5 times with PBS-T and incubated with goat F(ab')_2 _anti-human IgG (Fc)-horseradish peroxidase conjugate (Cappel-ICN Immunobiologicals, Costa Mesa, CA), diluted 1:2000 in incubation buffer, for 2 hours at room temperature. After washing 5 times with PBS-T, 100 μL of freshly made substrate (containing 0.5 mg/mL o-Phenylenediamine (Sigma) and 0.03% H_2_O_2 _in citrate/phosphate buffer, pH 4.9) was added to each well. After 10 minutes, the reaction was stopped with 50 μL 4N H_2_SO_4 _and absorbances were read at 490 nm. Results are expressed as anti-hHSP60 levels in arbitrary units (AU)/mL. Hereto, the mean OD of the triplicates was corrected for non-specific binding by subtraction of the mean OD in uncoated wells. Next, a standard curve was created by including in every assay a serial, 2-step dilution from 1:10 to 1:640 of a positive serum sample with anti-hHSP60 reactivity. The undiluted serum sample was arbitrarily assigned 100 AU/mL. Additionally, results are expressed as positive or negative. To establish the cut-off value for this test, the mean AU/mL and SD of the sera of 40 HC were determined, and the cut-off value was set at the mean + 2 SD.

For detection of antibodies against mHSP65, essentially the same procedure was followed. Recombinant mHSP65 (Stressgen) was diluted to 2 μg/mL and coated in a concentration of 0.1 μg/well. Serum was diluted 1:50 prior to incubation. To read results in AU, a standard curve was created as described for anti-hHSP60 antibodies. To establish the cut-off value for this test, the mean AU/mL and SD of the sera of 69 HC were determined, and the cut-off value was set at the mean + 2 SD.

### Myeloperoxidase inhibition assays

To test cross-reactivity between MPO and hHSP60, serum from one patient being positive for both MPO-ANCA and anti-hHSP60 antibodies was incubated for 30 minutes at 37°C with MPO or hHSP60 in concentrations of 1, 3, 10 and 20 μg/mL. The serum was then tested in the anti-hHSP60 ELISA as described above, or in the MPO-ANCA ELISA using commercially available direct ELISA kits (Euro-diagnostica, Malmö, Sweden). Additionally, absorption tests were performed in which ELISA plates were coated with hHSP60 or MPO and incubated with serum. The next day, or the second day, anti-hHSP60 reactivity in this pre-absorbed serum was tested as described above for the anti-hHSP60 ELISA. Unfortunately, this was the only patient of whom enough serum was present to perform the described tests.

### Statistical analyses

All data are presented as median [range] unless stated otherwise. Continuous data between groups were compared using Wilcoxon signed rank test and Kruskal-Wallis test with Dunn's post-test when appropriate. Due to the perceived relevance of the results, we did not only perform Dunn's post-test for subgroup analysis, but we also compared subgroups using Wilcoxon signed rank test. Correlations between data were derived using Spearman correlation. Odds ratios (OR) and 95% confidence intervals (CI) were obtained using Fisher's exact test. Analyses were performed with GraphPad Prism version 3.00 and GraphPad Instat software package version 2.04a (both GraphPad Software Inc., San Diego, CA). A two-sided p-value < 0.05 was considered to indicate statistical significance.

## Results

Eighty-six consecutive patients with AAV (44 PR3- and 42 MPO-ANCA) were included in the study. Mean age was 62.2 ± standard deviation 13.5 (65.9 ± 10.9 in MPO-ANCA and 59.7 ± 15.2 in PR3-ANCA, *P *= 0.07). Fifty-nine patients (69%) were male (27 patients (64%) in MPO-ANCA and 32 patients (73%) in PR3-ANCA, *P *= 0.49).

### Anti-mycobacterial HSP65 antibodies are elevated in MPO-ANCA compared to healthy controls

**Figure 1:**
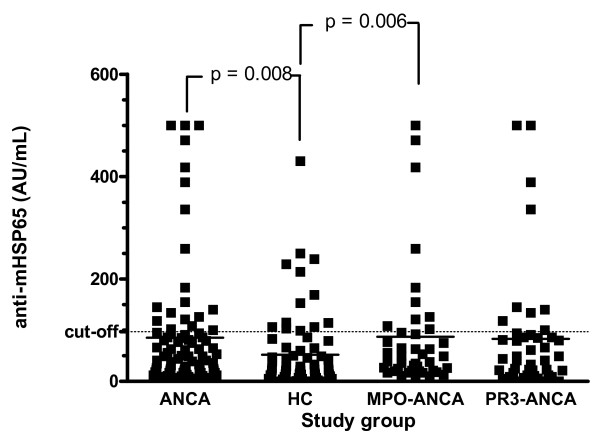
Anti-mHSP65 antibody levels in healthy controls (HC) and in patients with ANCA-associated vasculitis (AAV). Anti-mycobacterial Heat Shock Protein 65 (anti-mHSP65) antibody levels are significantly higher in AAV compared to HC (42.5 AU/mL [0–500] and 22 AU [0–430], respectively). Furthermore, anti-mHSP65 antibody levels in patients with PR3- and MPO-ANCA are shown. Anti-mHSP65 is comparable between patients with MPO-ANCA and PR3-ANCA (44 AU [7–500], and 42.5 AU [0–500], respectively). In patients with MPO-ANCA, anti-mHSP65 antibody level is significantly higher when compared to HC (*P *= 0.006). Dotted line represents cut-off value; solid line represents median value.

In patients with AAV, median anti-mHSP65 level was 42.5 AU/mL [0–500], compared to 22 AU/mL [0–430] in healthy controls (*P *= 0.008, figure 1). In MPO-ANCA, antibody levels were significantly higher (44 AU [7–500]) compared to HC (*P *= 0.006, figure 1). In patients with PR3-ANCA, there was a trend to a higher antibody level (42.5 AU [0–500] compared to HC (*P *= 0.10). With a cut-off value of 88.5 AU/mL, 13 of 69 HC (19%) were positive for anti-mHSP65 compared to 23 of 86 (27%) patients with AAV (*P *= NS). Eleven of 44 (25%) patients with PR3-ANCA were positive for anti-mHSP65 compared to 12 of 42 patients with MPO-ANCA (29%) (*P *= NS). Next, we confirmed the specificity of the anti-mHSP65 antibodies by immunoblotting. To this end we selected 5 sera that were positive by ELISA. All sera reacted with a 65 kD band in an immunoblot with recombinant mHSP65 (data not shown).

### Anti-human HSP60 antibodies are elevated in MPO-ANCA compared to PR3-ANCA and healthy controls

**Figure 2:**
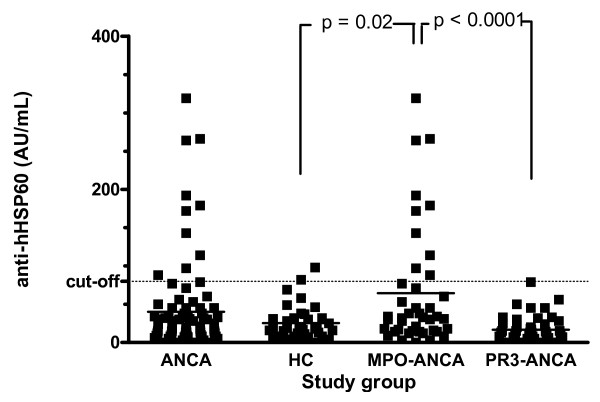
Anti-hHSP60 antibody levels in healthy controls (HC) and in patients with ANCA-associated vasculitis (AAV). Anti-human Heat Shock Protein 60 (anti-hHSP60) antibody levels are not significantly higher in AAV compared to HC (18 AU/mL [0–319] and 18.5 AU [0–98], respectively). Furthermore, anti-hHSP60 antibody levels in patients with PR3- and MPO-ANCA. Anti-hHSP60 are significantly higher in patients with MPO-ANCA compared to PR3-ANCA (32.5 AU [0–319], and 13 AU [0–79], respectively). In patients with MPO-ANCA, anti-hHSP60 antibody level is also significantly higher when compared to HC (*P *= 0.02), whereas in PR3-ANCA, it is not. Dotted line represents cut-off value; solid line represents median value.

In patients with AAV, median anti-hHSP60 level was 18 AU/mL [0–319], compared to 18.5 AU/mL [0–98] in healthy controls (*P *= NS, figure 2). In MPO-ANCA, antibody levels were significantly higher (32.5 AU [0–319]) compared to HC (*P *= 0.02) and PR3-ANCA (13 AU [0–79]) (*P *< 0.0001, figure 2). Patients with PR3-ANCA did not have higher antibody levels than HC. With a cut-off value of 80 AU/mL, 2 of 40 HC (5%) were positive for anti-hHSP60 compared to 12 of 86 (14%) patients with AAV (*P *= NS). One of 44 (2.3%) patients with PR3-ANCA was positive for anti-hHSP60 compared to 11 of 42 patients with MPO-ANCA (26%) (*P *= 0.001, OR 15.3, 95% CI 1.9 – 124). Next, we confirmed the specificity of the anti-hHSP60 antibodies by immunoblotting. To this end we selected 5 sera that were positive by ELISA. All sera reacted with a 60 kD band in an immunoblot with recombinant hHSP60 as well as with native bovine HSP60. The latter indicates that the antigenicity of recombinant and native HSP60 compares well (data not shown).

### Myeloperoxidase does not inhibit anti-hHSP60 antibody reactivity

**Figure 3:**
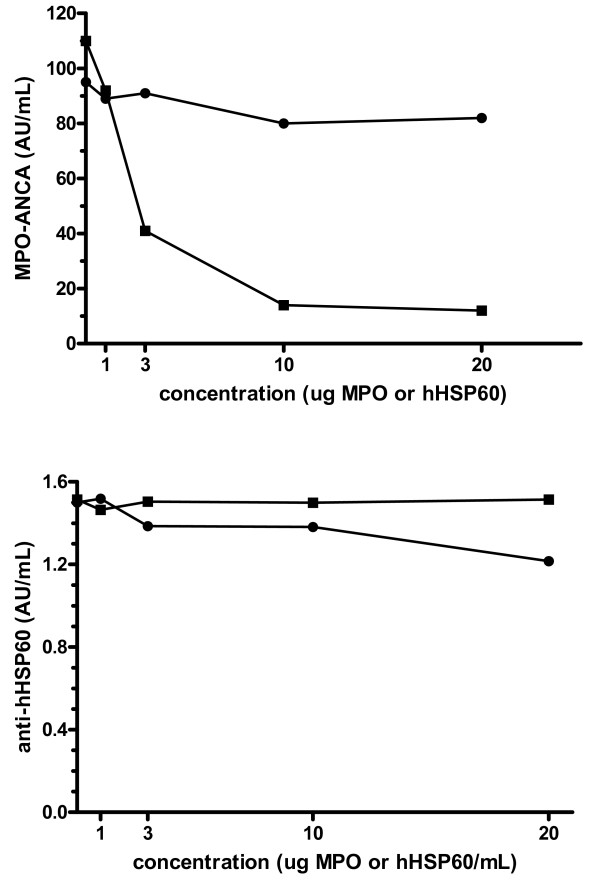
Upper panel : (3A) MPO-ANCA inhibition assay. Incubation with increasing concentrations of myeloperoxidase (MPO) (1 – 20 μg/mL) leads to a reduction in MPO-ANCA reactivity (■). In contrast, incubation with human Heat Shock Protein 60 (hHSP60) (1 – 20 μg/mL) does not decrease MPO-ANCA reactivity (●). Lower panel: (3B) Anti-human Heat Shock Protein 60 (hHSP60) inhibition assay. Incubation with increasing concentrations of myeloperoxidase (MPO) (1 – 20 μg/mL) does not lead to a reduction in anti-hHSP60 reactivity (■). However, incubation with hHSP60 (1 – 20 μg/mL) only slightly decreases anti-hHSP60 reactivity (●)

**Figure 4:**
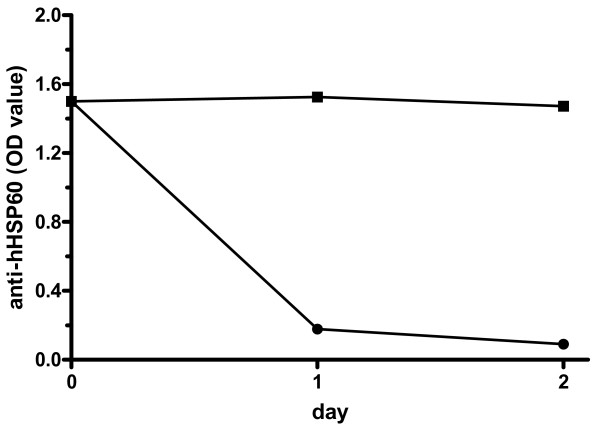
MPO and hHSP60 absorption assays. Incubation of serum in ELISA plates coated with human Heat Shock Protein 60 (hHSP60) for 1 or 2 days leads to a reduction in anti-hHSP60 reactivity (●). In contrast, incubation of serum in ELISA plates coated with myeloperoxidase (MPO) does not decrease anti-hHSP60 reactivity (■).

To test whether anti-hHSP60 antibodies actually cross-react with MPO, we performed inhibition assays incubating serum from the patient with the highest anti-hHSP60 response with MPO or hHSP60 and testing for hHSP60 and MPO reactivity. Incubation with increasing concentrations of MPO inhibited the MPO-ANCA response in a dose-dependent manner (figure 3A). However, incubation of serum with hHSP60 did not inhibit the MPO-ANCA reactivity (figure 3A). Additionally, MPO was unable to inhibit the anti-hHSP60 antibody reactivity (figure 3B). However, incubation with hHSP60 did not inhibit the anti-hHSP60 response (figure 3B). To test for the ability of hHSP60 to inhibit anti-hHSP60 reactivity, absorption tests were performed. Results now showed inhibition of the anti-hHSP60 response by hHSP60 but not by MPO (figure 4).

### Follow-up anti-hHSP60 levels

**Figure 5:**
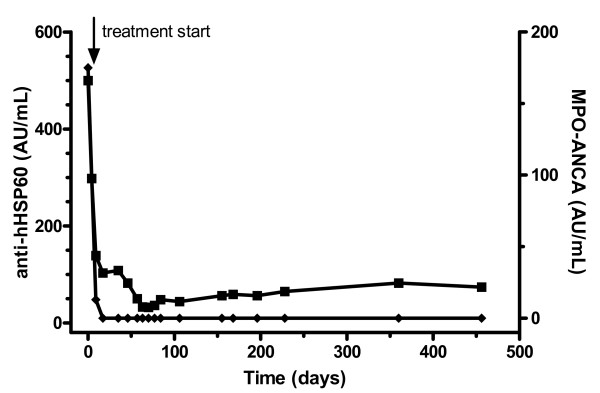
Follow-up of a patient positive for antibodies against human Heat Shock Protein 60 (anti-hHSP60). Anti-hHSP60 levels (■) drop as well as antibody levels against myeloperoxidase (MPO-ANCA) (◆) once treatment has commenced, but increase during follow-up, while MPO-ANCA levels remain undetectable.

To further exclude cross-reactivity between hHSP60 and MPO-ANCA, we tested whether anti-hHSP60 antibody levels were only present if there was presence of MPO-ANCA. In the patient with MPO-ANCA and the highest anti-hHSP60 level, follow-up serum samples were available to test for the presence of anti-hHSP60. As shown in figure 5, anti-hHSP60 levels dropped analogous to MPO-ANCA levels once treatment has commenced, but slightly increased with tapering of the treatment while MPO-ANCA levels remained undetectable.

### Correlation between anti-mHSP65 and anti-hHSP60 but not between MPO-ANCA and anti-hHSP60 antibodies

Finally, we checked whether correlation exists between antibody levels against mHSP65, hHSP60, and MPO. There was a correlation between height of antibody levels against mHSP65 and hHSP60 (Spearman r = 0.32, *P *= 0.003), but no correlation existed between MPO-ANCA and anti-hHSP60 (Spearman r = -0.064, *P *= 0.69). Also, there was no correlation between anti-mHSP65 and MPO-ANCA (Spearman r = 0.074, *P *= 0.65).

## Discussion

The mechanism by which ANCA are induced are as yet unclear. One favored hypothesis is that infections may trigger an ANCA response [11]. We hypothesized that infections may trigger the ANCA response against MPO through hHSP60. We found that anti-hHSP60 was almost exclusively found among patients with MPO-ANCA when compared to PR3-ANCA, supporting our hypothesis. Nevertheless, there was no correlation between MPO-ANCA and anti-hHSP60 antibody levels, and inhibition and absorption assays did not show cross-reactivity between hHSP60 and MPO-ANCA. Furthermore, when we determined the anti-mHSP65 response in AAV and HC, we found no difference between patients with MPO-ANCA and patients with PR3-ANCA, and only a weak correlation between anti-hHSP60 antibody levels and anti-mHSP65 levels. However, not every patient who was positive for anti-hHSP60 had antibodies against mHSP65, suggesting that anti-hHSP60 and anti-mHSP65 are not the same antibodies, and that an anti-hHSP60 response may not be triggered simultaneously with an anti-mHSP65 response.

Increased anti-HSP antibody levels, as observed in the present study, might be artificial because of two reasons. First, patients with several systemic autoimmune diseases are known to have increased levels of circulating IgG and this might result in a simultaneous increase in antibodies to, for instance, HSP60 and HSP65. Although analysis of IgG levels is not a standard procedure for the diagnostic work-up of an AAV patient, IgG data were available for a subset of patients included and all were within the normal range (data not shown). Furthermore, to our knowledge such hypergammaglobulinaemia has not been described for AAV. Second, since healthy controls in the current study were of lower age than the AAV patients, increased levels of antibodies to HSP60 and HSP65 might be due to ageing. Again this is not very likely since there was no correlation whatsoever (R^2 ^< 0.1) between antibody titers and age (data not shown). Therefore we conclude that antibody levels to HSP60 and HSP65 are really increased in AAV patients as compared to healthy controls.

The shared sequence homology described between hHSP60 and MPO is a theoretical one, based on amino acid sequence. This sequence similarity has a length of 17 amino acids and is present on the heavy chain of myeloperoxidase [15]. The sequence is present in a region of myeloperoxidase that is recognized as an epitope by MPO-ANCA in some patients [19]. However, it is unknown whether anti-hHSP60 antibodies bind to the corresponding amino acid sequence of hHSP60. To test whether anti-hHSP60 antibodies actually cross-react with MPO, we performed inhibition assays incubating serum from the patient with the highest anti-hHSP60 response with MPO or hHSP60 and testing for hHSP60 and MPO reactivity. We could not detect a decrease in hHSP60 reactivity when serum was incubated with MPO, suggesting that although sequence homology exists, epitopes recognized by MPO-ANCA are different from those recognized by anti-hHSP60 antibodies. Obviously, this test cannot completely exclude that cross-reactivity exists in other patients, although the absence of correlation between MPO-ANCA and anti-hHSP60 also does not support such cross-reactivity.

Antibodies against hHSP60 are associated with the presence and severity of atherosclerosis [20-22], although this has been disputed by others [23]. Interestingly, we found elevated levels of anti-hHSP60 antibodies only in patients with MPO-ANCA. An explanation for this finding may be that diagnosis of MPO-AAV is often delayed, and this may lead to prolonged inflammation and eventually to elevated levels of anti-hHSP60 in MPO-ANCA patients when compared to PR3-ANCA patients. Our data might indicate that cardiovascular disease is more often present in patients with MPO-ANCA. However, follow-up data in our small study do not confirm this.

Interestingly, anti-hHSP60 reactivity could not be inhibited by our first inhibition assay. This may be due to the nature of the inhibition assay. In the first assay, hHSP60 was added to the serum of the anti-hHSP60 positive patient, whereas in the second assay, hHSP60 was coated to the ELISA plate. This second assay clearly demonstrated inhibition of the anti-hHSP60 response. An explanation for this phenomenon may be that anti-hHSP60 antibodies are unable to bind to fluid phase hHSP60, but can bind to solid phase hHSP60, as demonstrated by the second inhibition assay, and the positive antibody response demonstrated in the anti-hHSP60 ELISA.

## Conclusion

We have shown that antibodies against hHSP60 are elevated in patients with MPO-ANCA in comparison with PR3-ANCA. These findings suggest a role for infections in the pathogenesis of MPO-AAV through molecular mimicry between bacterial HSP65, human HSP60 and MPO. Additional absorption assays, however, did not support this hypothesis. Whether there is a relationship between anti-hHSP60 and cardiovascular disease in patients with MPO-ANCA remains to be studied.

## Competing interests

The author(s) declare that they have no competing interests.

## Authors' contributions

MCS participated in the design and coordination of the study, performed the statistical analysis and drafted the manuscript. RT carried out the various assays and helped to draft the manuscript. PvP participated in the coordination of the study and revised the manuscript critically. JGMCD participated in the design of the study and helped to draft the manuscript. JWCT conceived of the study, participated in its design and helped to draft the manuscript.
